# CRISPR-based large-scale modeling of loss-of-function mutations to investigate mechanisms of stress resistance in cancer

**DOI:** 10.1016/j.xpro.2023.102097

**Published:** 2023-02-11

**Authors:** Fabrizio Simeoni, Ioannis Loukas, Thomas Stuart Wilson, Paola Scaffidi

**Affiliations:** 1 Cancer Epigenetics Laboratory, The Francis Crick Institute, NW1 1AT London, UK; 2 Department of Experimental Oncology, IEO, European Institute of Oncology IRCCS, 20139 Milan, Italy

**Keywords:** Cell Biology, Cell-based Assays, Cancer, Genetics, High-throughput Screening, Molecular Biology, CRISPR

## Abstract

Dissecting mechanisms driving subclone expansion in primary cancers has been challenging. Here, we present a protocol to systematically disrupt entire gene networks and assess the functional impact of this perturbation on cancer cell fitness. By combining arrayed CRISPR libraries and high-content microscopy, we describe steps to identify classes of genes whose inactivation promotes resistance to environmental challenges faced by cancer cells during tumor growth or upon therapy. A proof-of-principle interrogation of the epigenetic regulatory network is described.

For complete details on the use and execution of this protocol, please refer to Loukas et al. (2022).^1^

## Highlights

• Large-scale modeling of loss-of-function mutations selected in human cancers
• Generation of arrayed KO populations via sgRNAs transduction or crRNAs transfection
• Analysis of network-level perturbation on cancer cell fitness under stress

## Abstract (editor highlights)

Publisher’s note: Undertaking any experimental protocol requires adherence to local institutional guidelines for laboratory safety and ethics.

## Graphical abstract

**Figure:**
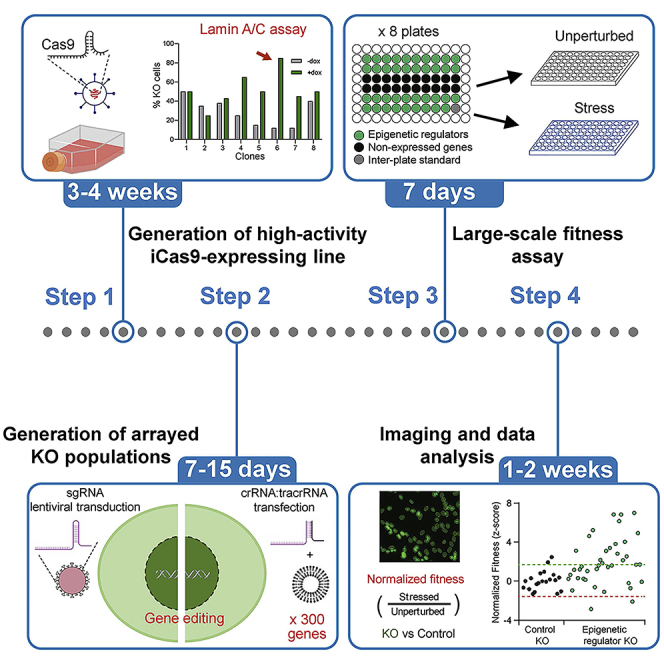

## Before you begin

A major challenge in cancer biology is the characterization of genotype-phenotype relationships. To date, approximately 2,000 genes affected by mutations in cancer patients have been implicated in the disease.^2^ Yet, functional investigation has only focused on a small subset of these, and even for validated cancer genes, our understanding of how their disruption promotes the disease is limited. In this protocol, we describe how to model loss-of-function mutations in hundreds of genes with putative tumor-protective functions, and perform high-throughput functional assays to characterize their impact on cell fitness in unfavorable conditions. This approach is particularly valuable when mutations in functionally-related genes may confer similar phenotypes. We have recently applied this method to interrogate over 300 epigenetic regulator genes in cancer cells, mimicking the frequent inactivating mutations that affect the epigenetic regulatory network in cancer patients, especially in emerging subclones.^1^ After generation of epigenetically-disrupted cells, we illustrate how to assess the impact of this network-level perturbation on cancer cell fitness under various types of stress that are typical of the tumor microenvironment.

While selection-based assays employing pooled single-guide RNA (sgRNA) libraries identify genes whose perturbation leads to the strongest phenotype under the experimental conditions used, high-throughput assays using arrayed libraries allow systematic interrogation of all genes, or specific gene classes, detecting both strong and milder phenotypes. Furthermore, high-throughput assays provide more flexibility in terms of functional readout: in addition to measurements of cell fitness, described here, numerous readouts based on immunofluorescence, live cell imaging, luminescence, or colorimetric assays can be used. Due to the high sensitivity and flexibility, this approach offers a powerful means to systematically disrupt entire gene networks and assess the impact of loss-of-function mutations on different aspects of cell biology.

Here, we describe the steps required to systematically disrupt the epigenetic network in cells derived from a patient-derived-xenograft (PDX) model of melanoma (MEXF 2090) and assess the fitness of cancer cells under various stressful conditions, from CRISPR library design to data analysis. The protocol details steps for generating arrayed knock-out (KO) cell populations using either lentiviral constructs expressing sgRNAs or chemically synthesized guide RNAs.

### Sourcing an arrayed guide RNA library

**Timing: 1 day**

CRISPR-Cas9 has become the primary tool for gene editing and a variety of reagents have been made available to the research community. In this section, we introduce two options for large-scale generation of KO populations using arrayed guide RNAs in 96-well format: transduction of sgRNAs and transfection of complexes of synthetic crispr RNA (crRNA) and trans-activating crRNA (tracrRNA). Neither approach is universally better, with cell type and target genes affecting performance, and both methods presenting advantages and disadvantages. Thus, the choice of which method to use mainly depends on the expertise of the laboratory, the resources available and the scale of the experiment. sgRNAs are generally introduced in cells via lentiviral transduction, which enables selection of infected cells and sustained transcription of sgRNAs for efficient KO. Integration of the lentiviral construct in the genome may interfere with some applications, such as investigation of mechanisms regulating genome maintenance. crRNA-tracrRNA complexes are instead transfected and are quickly cleared from cells, avoiding potential deleterious effects of exogenous DNA integration in the genome. Editing efficiency is highly dependent on the transfection reagent used and pilot experiments are necessary to establish optimal transfection conditions. Transfected cells cannot be selected due to the transient nature of the method. Generation or propagation of lentiviral sgRNA libraries requires additional steps as described in ours and others’ studies,^3^^,^^4^ but allow performance of unlimited experiments; synthetic crRNAs are easier to source and use, but support a limited number of experiments and the cost of purchase is relatively high.

#### Lentiviral sgRNA library

The library used in this protocol is sourced from a focused lentiviral library targeting epigenetic or transcriptional regulators and proteins involved in the DNA damage response. Each gene is targeted by 7 sgRNAs.^3^ From the available library, constructs targeting 318 genes, encoding stricto-sensu epigenetic regulators, were chosen.^1^^,^^3^ If other functional gene classes need to be targeted alternative sources are available. As an example, Sigma-Aldrich and the Wellcome Trust Sanger Institute have joined forces and created genome-wide arrayed libraries for both mouse and human studies. Each library contains two optimized sgRNAs for each gene, covering 17,000 human and over 20,000 mouse genes in total. Moreover, the company offers focused arrayed libraries targeting entire networks, such as Proteases, Transcription Factors, Phosphatases, Transcription Factors, Ubiquitin Enzymes, GPCRs, cell cycle proteins and Drug Targets.

#### Synthetic crRNA library

Focused Edit-R crRNA libraries can be purchased or assembled from off-the-self reagents available at Horizon discovery. The company offers pre-made libraries targeting different human or mouse networks such as Proteases, Protein Kinases, Phosphatases, Transcription Factors, Ubiquitin Enzymes, GPCRs, Ion Channels and Drug Targets. Moreover, a cherry-pick library option is available (https://horizondiscovery.com/en/ordering-and-calculation-tools/cherry-pick-library-loader), where crRNAs of interest can be selected by uploading a gene list, and organized in the desired layout for customized plates. Individual or pools of four synthetic crRNAs can be chosen for each target gene.

### Plate layout in 96-well format

**Timing: 1 day**

Depending on the scale of experiment, guide RNAs may need to be arranged in multiple plates, with each one containing negative controls and an inter-plate standard. When assessing the effects of stress on cellular fitness, experiments must be carefully controlled to account for both technical and biological variability. For instance, minor differences in number or distribution of seeded cells can have a detectable effect on the observed phenotype. Furthermore, the cellular response to stress has a stochastic component, which leads to intrinsically variable phenotypes across replicates. Thus, multiple negative control wells are required to account for these sources of noise. The most stringent negative control is targeting of a non-expressed gene, so that all stages of the cellular response to CRISPR/Cas9 induced gene editing are accounted for. Transcriptomic analysis or targeted qRT-PCR can be used to identify suitable genes, that are not expressed both in unperturbed and in stress conditions. For example, *TNP1*, *TNP2*, *HMGB4*, *SMC1B* and *DNMT3L* were determined to be unexpressed in MEXF 2090 cells and were used as negative control in our study.^1^ To target 318 epigenetic regulator genes the library is arranged in eight 96-well plates, each containing sgRNAs targeting 40 expressed genes, the 5 non-expressed genes (4 replicates each for a total of 20 negative controls) and one inter-plate standard targeting the same expressed gene, which monitors the consistency in experimental conditions across plates. It is essential to exclude external wells and fill with PBS to avoid edge effects which strongly affect cell fitness (Graphical Abstract – step 3).

### Titration of environmental stressors

**Timing: 4–5 days**

It is important to test cultured cells in diverse unfavorable contexts and monitor their growth kinetics over time. This allows optimization of experimental conditions and assay duration. To determine optimal stress conditions for the large-scale fitness assay, dose-response curves for each stressor are generated using the Incucyte® S3 Live-Cell Analysis System. While the system allows live cell imaging for kinetic analysis, end-point measurements are more practical when testing multiple conditions. Here we describe modelling of three types of environmental challenges faced by cancer cells during cancer evolution: reduced nutrient availability (glutamine deprivation), acidification of the microenvironment, a frequent consequence of hypoxia in tumors (reduced pH induced by HCl addition) and drug treatment (Buparlisib, PI3K Inhibitor).

As a general guideline, conditions that reduce cell fitness by 40%–60% over a 2-day treatment are considered optimal for the large-scale assay as they create a window of opportunity to detect enhanced or reduced fitness phenotypes, upon generation of KO populations.

**CRITICAL:** Once the first titration has been made, we recommend validating the chosen condition in an independent experiment before starting the screen. Since not all stressors affect cell viability in a similar manner and the cellular response to stress is highly variable depending on the type of stress applied, in-depth characterization is required. For example, decreasing doses of L-Glutamine in the medium leads to minimal effects on MEXF 2090 cell fitness, only showing a stressed phenotype when it is completely removed. Instead, Buparlisib treatment shows a linear dose-response. Moreover, while both of the above stated stressors are sustained throughout the length of the assay, low pH is tolerated only as a transient stressor and physiological pH needs to be restored at day 3, after the initial cellular response, to avoid death across the whole cell population.

***Note:*** Most steps of this protocol can be executed using a multichannel pipette to reduce the time of pipetting and to minimize technical mistakes.

**CRITICAL:** Excessively harsh conditions will result in widespread cell death, lowering the chance for cell adaptation to the stress. Mild conditions will instead reduce the dynamic range of the assay. Therefore, it is essential to titrate the source of the stress to find the optimal window. Conditions that reduce cell fitness by 40%–60% are considered optimal for the large-scale assay.

1. Day 1: Seed cells in 96-well plates.
  a. Culture MEXF 2090 cells (doubling time: 21 h) in complete RPMI medium containing 10% fetal bovine serum (FBS), 1% L-Glutamine and 1% Pen/Strep.
  b. Take one 10-cm dish of healthy cells (70%–80% confluency), trypsinize and count cells.
  c. Seed MEXF 2090 cells at 2,000–3,000 cells per well in a 96-well plate format.
    ***Note:*** The seeding density may need to be adjusted for a different cell line. The treatment needs to be stopped once the untreated cell population reaches confluency; therefore, the goal is to have enough time for cells to show a response to the environmental stress applied. Cells should be seeded at least in triplicate for each condition. MEXF 2090 cells are a very fast-growing cell line and will reach confluency in 48 h.
    i. Calculate the necessary volume of the cell suspension for 2,000–3,000 cells per well. 100 μL of cell suspension per well is enough in a 96-well plate.
    ii. Add the calculated volume of cells into fresh medium in a conical tube. Mix thoroughly by pipetting.
    iii. Pipette 100 μL of cell suspension in each well. Place plates back in the incubator to allow cells to attach (12–16 h).
      **CRITICAL:** Avoid using the outer wells and fill with 200 μL of PBS.

2. Day 2: Start culture in stressful conditions.
  a. Design the plate layout such that different doses of stressors can be tested simultaneously.
    ***Note:*** The range of concentrations is determined empirically or by doing a literature research. L-glutamine, HCl and PI3K inhibitors (Buparlisib) are all titrated within the following range: L- Glutamine (0%–1%), acidity (pH 6.0–6.7) and Buparlisib (1 nM - 1μM).
  b. Prepare serial dilutions of the stressors with a fixed dilution factor.
    i. Prepare several tubes containing fresh medium in conical tubes.
    ii. Prepare the highest concentration from the stock solution in the first conical tube, mixing well by inverting or vortexing.
    iii. Transfer the required volume to the second tube, mixing.
    iv. Repeat the same procedure until the lowest concentration is made.
    v. Also prepare a tube for untreated control by adding the same volume of vehicle solution.
  c. Collect cells from the incubator and discard medium by aspirating.
  d. After washing cells with PBS, dispense the prepared medium into each well (100 μL per well) and return cells in the incubator.

3. Day 4–5: Assess cell fitness with Incucyte® S3 Live-Cell Analysis System.
  ***Note:*** Any other system able to scan 96-well plates with a microscopy-based readout can be used instead of the Incucyte® S3 Live-Cell Analysis System.
  a. Prepare enough volume of fixing solution (4% paraformaldehyde (PFA) in PBS) and of staining solution (0.5% Triton-X in PBS containing 1: 4,000 SYTOX Red or 1: 20,000 SYTOX Green).
    ***Note:*** SYTOX Red or Green can be used interchangeably.
  b. Retrieve the cells from the incubator. Discard the medium by inverting the plate.
  c. Wash the cells with 100 μL of PBS and eliminate PBS by inverting the plate.
  d. Fix cells using 100 μL of 4% PFA in PBS for 15 min. Pipette gently and to the wall of the well to avoid cell detachment.
  e. Eliminate PFA by inverting the plate and wash cells twice with PBS (100 μL per well).
  f. Permeabilize cells using 100 μL of 0.5% Triton-X in PBS for 10 min at 20°C–25°C containing SYTOX Red or Green.
  g. Wash the cells with 100 μL of PBS twice and image with Incucyte® S3 Live-Cell Analysis System using the appropriate fluorescent channel.

4. Data analysis (For comprehensive description, see imaging and data analysis section below).
  a. Apply a mask by thresholding fluorescent nuclei and count the number of objects.
  b. Calculate the relative cell fitness by dividing the number of treated cells by the count of untreated cells.
  c. Chose conditions that result in 40%–60% reduction in cell counts for the final screen.

**CRITICAL:** If the cells are semi-adherent or easily lift such as HEK293T cells, you can fix by adding 2× PFA (8%) directly into the medium. Do not pipet up and down, it will mix by itself.

### pLenti-sgL*MNA*-GFP virus production

**Timing: 3–4 days**

Efficient gene editing, which is required for accurate comparison of numerous KO populations in functional assays, can be achieved by optimizing Cas9 activity in cells. The following steps describe how to produce lentiviral particles for transduction of a validated sgRNA, which will be used to identify suitable Cas9-expressing monoclonal populations.

5. Day 1: Plate HEK293T cells for lentiviral production.
  a. Culture HEK293T cells in complete DMEM medium containing 10% FBS, 1% L-Glutamine, and 1% Pen/Strep.
  b. Harvest cells from a medium flask, count cells, and seed 2 million cells in a 10-cm dish. This number will achieve 80%–90% confluency the following day.
  c. Incubate for 12–16 h at 37°C with 5% CO_2_.

6. Day 2: HEK293T transfection.
  a. Thaw the plasmids, allow FuGene HD to reach 20°C–25°C, and warm cell culture medium and OptiMEM to 37°C.
  b. For pLenti-sgLMNA-GFP, prepare lentiviral plasmid mix for transfection as below:
    ***Note:*** HEK293T cells can be transfected with alternative reagents such as Lipofectamine, Calcium and Polyethylenimine. In our experience, FuGene HD yields the highest transfection efficiency.

**Table:** 

Reagent	Amount
Lentiviral construct (pLenti-sgLMNA-GFP)	5 μg
psPax2 (packaging)	3.75 μg
pMD2G (VSVG envelope)	1.25 μg
Optimem	Up to 500 μL
  c. Add 30 μL of FuGene HD to achieve the required 3:1 ratio of Reagent (μL) : DNA (μg), mix well and incubate at 20°C–25°C for 15 min.
    **CRITICAL:** Do not allow FuGene HD to touch the wall of the tube (as indicated by the manufacturer protocol), add directly to the medium.
  d. Replace the HEK293T medium, using 25% lower volume than the standard to increase lentiviral concentration.
  e. After incubation, add the Fugene-DNA mixture dropwise.
  f. Incubate for 12–16 h at 37°C with 5% CO_2_.

7. Days 3–4: Harvest lentiviral-containing medium.
  a. Recover the supernatant containing the viral particles, and pass through a 0.45 μL filter.

***Note:*** This step can be done 24 h post transfection and repeated at 48 h for the second batch of virus. Virus can be aliquoted and stored at −80°C if needed.

## Key resources table

**Table:** 

REAGENT or RESOURCE	SOURCE	IDENTIFIER
**Antibodies**		

LaminA/C (dilution: 1:200)	Santa Cruz	Cat#sc-7292, RRID: AB_627875
anti-mouse-568 secondary antibody (dilution: 1:400)	Invitrogen	Cat#A10037, RRID: AB_2534013

**Biological samples**		

Patient Derived Xenograft MEFX 2090	Charles Rivers Tumor Model Compendium	https://compendium.criver.com/

**Chemicals, peptides, and recombinant proteins**		

FuGENE® HD Transfection Reagent	Promega	Cat#E2311
5× siRNA buffer	Horizon	Cat#B-002000-UB-100
DharmaFECT™ Duo Transfection Reagent	Horizon	Cat#T-2010-01
Paraformaldehyde, 16% w/v aq. soln., methanol free	Alfa Aesar	Cat#43368
SYTOX™ Green Nucleic Acid Stain	Thermo Fisher Scientific	Cat#S7020
SYTOX™ Deep Red Nucleic Acid Stain	Thermo Fisher Scientific	Cat#S11380
Buparlisib	Generon	Cat#HY-70063
Polybrene	Santa Cruz	Cat#sc-134220
Triton™ X-100	Merk	Cat#9036-19-5
Lullaby	OZBiosciences	Cat#LL70500
DharmaFECT Set of 4 Transfection Reagents	Horizon	Cat# T-2005-01
DharmaFECT Duo	Horizon	Cat#T-2010-01
Lipofectamine RNAiMAX	Thermo Fisher	Cat#13778100
Fetal bovine serum	Thermo Fisher Scientific	Cat#10270106
Penicillin-Streptomycin	Merck	Cat#P4333
L-Glutamine solution	Merck	Cat# G7513
DAPI	Merck	Cat#D9542

**Critical commercial assays**		

MultiScreenHTS HV Filter Plate, 0.45 μm, clear, sterile	Millipore	Cat#MSHVS4510
Nunc™ Seals, Tapes, and Foils	Thermo Fisher Scientific	Cat#232698

**Deposited data**		

Z-score from large-scale fitness assays	Loukas et al.^1^	Table S3 from Loukas et al.^2^

**Experimental models: Cell lines**		

PDX MEFX 2090 derived cells	Loukas et al.^1^	Table S2 from Loukas et al.^2^
Cas9 - expressing MEFX 2090 cells	Loukas et al.^1^	N/A
HEK293T	Francis Crick Institute Cell Line Repository	N/A

**Oligonucleotides**		

Synthetic crRNAs used to generate KO cells	Loukas et al.^1^	Table S7 from Loukas et al.^2^

**Recombinant DNA**		

Plasmid: pCW-Cas9	Monserrat et al.^5^	N/A
Plasmid: psPax2	Trono Lab	Addgene; Cat#12260; RRID: Addgene_12260
Plasmid: pMD2.G	Trono Lab	Addgene; Cat#12259; RRID: Addgene_12259
Plasmid: pAdVAntage™ Vector	Promega	Cat#E1711
Plasmid: arrayed lentiviral sgRNA library	Henser-Brownhill et al.^3^ Loukas et al.^1^	Table S2 from Henser-Brownhill et al.^3^ Table S3 from Loukas et al.^1^
Plasmid: pLenti-sgLMNA-GFP (GGCGAGCTGCATGATCTGCG)	N/A	N/A

**Software and algorithms**		

Incucyte® S3 Live-Cell Analysis System	Sartorius	RRID: SCR_019874; https://www.sartorius.com/en/products/live-cell-imaging-analysis/live-cell-analysis-instruments
Prism 9	GraphPad Software	RRID: SCR_002798; https://www.graphpad.com/

**Other**		

RPMI 1640 Medium, HEPES	Thermo Fisher Scientific	Cat#22400089
RPMI 1640 Medium, no glutamine	Thermo Fisher Scientific	Cat#21870076
DMEM high glucose pyruvate, no glutamine	Thermo Fisher Scientific	Cat#21969035

## Step-by-step method details

### Screening of iCas9-expressing clones by editing activity

**Timing: 3–4 weeks**

The success of the large-scale fitness assay depends on the ability to generate KO populations efficiently and reproducibly across wells. Upon transduction of doxycycline-inducible Cas9 (iCas9) in the cell line of interest following standard procedures,^5^^,^^6^ a clonal line is required to ensure homogenous editing.

**CRITICAL:** The editing efficiency observed with iCas9 is highly variable across a polyclonal population. Moreover, leaky Cas9 expression can be detected in the absence of doxycycline (dox). Therefore, a clone exhibiting high editing efficiency and minimal editing prior to induction needs to be isolated. Since Cas9 mRNA or protein levels in isolated clones rarely correlate with activity, we have designed a simple assay to screen clones based on editing efficiency. The strategy employs a validated sgRNA targeting a non-essential and broadly expressed gene, *LMNA*, which is transduced into isolated clones*.* Subsequent detection of the encoded LaminA/C protein through a highly-specific antibody enables rapid quantification of KO efficiency in multiple clones in parallel using immunofluorescence and high-content microscopy.

***Note:*** This protocol starts after the transduction of doxycycline-inducible Cas9 (iCas9) in MEXF 2090 cells using the pCW-Cas9-Hygro lentiviral construct and selection of infected cells by hygromycin treatment, following standard procedures.^5^

***Note:*** We recommend infecting at least 5–10 million cells as transduction of iCas9 is highly inefficient.

1. Day 1: Isolating single cells by flow cytometry cell sorting.
  a. Harvest and resuspend hygromycin-resistant, Cas9-expressing MEXF 2090 cells in FACS buffer.
  b. Use an appropriate gating strategy for your FACS machine to stringently sort for single cells, and sort them one-per-well into the prepared 96-well plates. Single cells are sorted using side and forward scatter.

***Note:*** While using a FACS machine is the easiest way of deriving clean single-cell populations, other methods of clonal isolation such as dilution plating or transfer of grown colonies after selection are possible, and could be used instead.^7^

2. Day 2–20: Growing and maintaining clones.
  ***Note:*** The time taken for a clonal line to reach confluency is variable by cell type, but usually takes 2–3 weeks.
  a. Change the media in each plate of clones twice a week.
  b. Begin looking for successful clones after ten days. The recovery rate of clonal lines is variable according to the cell type and sorting conditions, but it is usually around 5%–15% of sorted cells.

3. Day 21–26: Seeding Cas9 clones for functional assay.
  a. When most clones on the plate are approaching confluency, collate together into three replicate plates. Two plates are for the *LMNA*-editing assay (+ and – doxycycline). One replicate is kept for subsequent expansion of suitable clones.

4. Transduction of *LMNA*-targeting sgRNAs.
  a. Prepare the pLenti-sgLMNA-GFP lentivirus (as described in the section: pLenti-sgLMNA-GFP virus production).
  b. Dilute virus 1:1 in fresh medium containing 5 μg mL^-1^ polybrene.
  c. Add this to both plates, at 100 μL per well. Incubate the plates for 12–16 h.
    ***Note:*** The required concentration of polybrene can vary, and should be optimised for other cell lines.
  d. The following day, replace the cell culture medium. On one plate, add medium only, and on the other add medium containing 1 μg mL^−1^ doxycycline. Incubate for 72 h.
    ***Note:*** Lamin A/C has a half-life of approximately 24 h and cell division is required for degradation of pre-existing protein.
    ***Note:*** Slow-cycling cell lines may require longer incubation prior to the next step, to ensure reliable detection of KO cells.

5. LaminA/C immunofluorescence.
  a. Prepare the required solutions for immunofluorescence.
    i. Fixation solution: 4% paraformaldehyde (PFA) in PBS.
    ii. Permeabilization buffer: 0.5% Triton X in PBS.
    iii. Blocking buffer: 3% Bovine Serum Albumin and 0.05% Triton X in PBS.
  b. At 96 h after infection, fix cells with 100 μL of fixation solution per well, incubating at 20°C–25°C for 15 min.
    **CRITICAL:** Fixation conditions are critical for reproducible immunofluorescence, so ensure that the dilution and incubation are both accurate.
  c. Discard PFA, wash three times with PBS (5 min each), and permeabilize with 100 μL of 0.5% Triton X in PBS for 5 min.
  d. Remove the permeabilization solution by inverting the plate and add 50 μL blocking buffer per well. Incubate at 20°C–25°C for 30 min.
  e. Dilute the primary antibody 1:200 in blocking buffer.
  f. Remove the blocking buffer and add 50 μL of primary antibody dilution per well. Incubate at 20°C–25°C for 30 min.
  g. Remove the primary antibody by inverting the plate, and wash three times with PBS.
  h. Dilute the anti-mouse-568 secondary antibody 1:400 in blocking buffer.
  i. Remove the PBS, and add 50 μL per well. Incubate at 20°C–25°C for 30 min, shielding from light.
  j. Remove the secondary antibody, add PBS containing 1.5 μg mL^-1^ of DAPI and incubate for 5 min.
  k. Wash three times with PBS and seal plates.

6. Analysis of editing efficiency of infected Cas9 clones.
  a. Image cells using a high-content microscope, such as a Thermo Scientific ArrayScan VTI HCS Reader, equipped with standard filter sets for DAPI, FITC, and TRITC.
  b. Analyze images with the following pipeline:
    i. Identify nuclei using DAPI.
    ii. Select GFP + nuclei using the FITC channel.
    iii. Measure LaminA/C levels in GFP+ and GFP- nuclei using the TRITC channel.
    iv. Set a threshold of LaminA/C signal such that less than 5%–10% of cells in the control, uninfected population are below that value.
    v. Quantify LaminA/C-negative cells, with fluorescent signal below the threshold value, in GFP+ nuclei.

**Figure 1:**
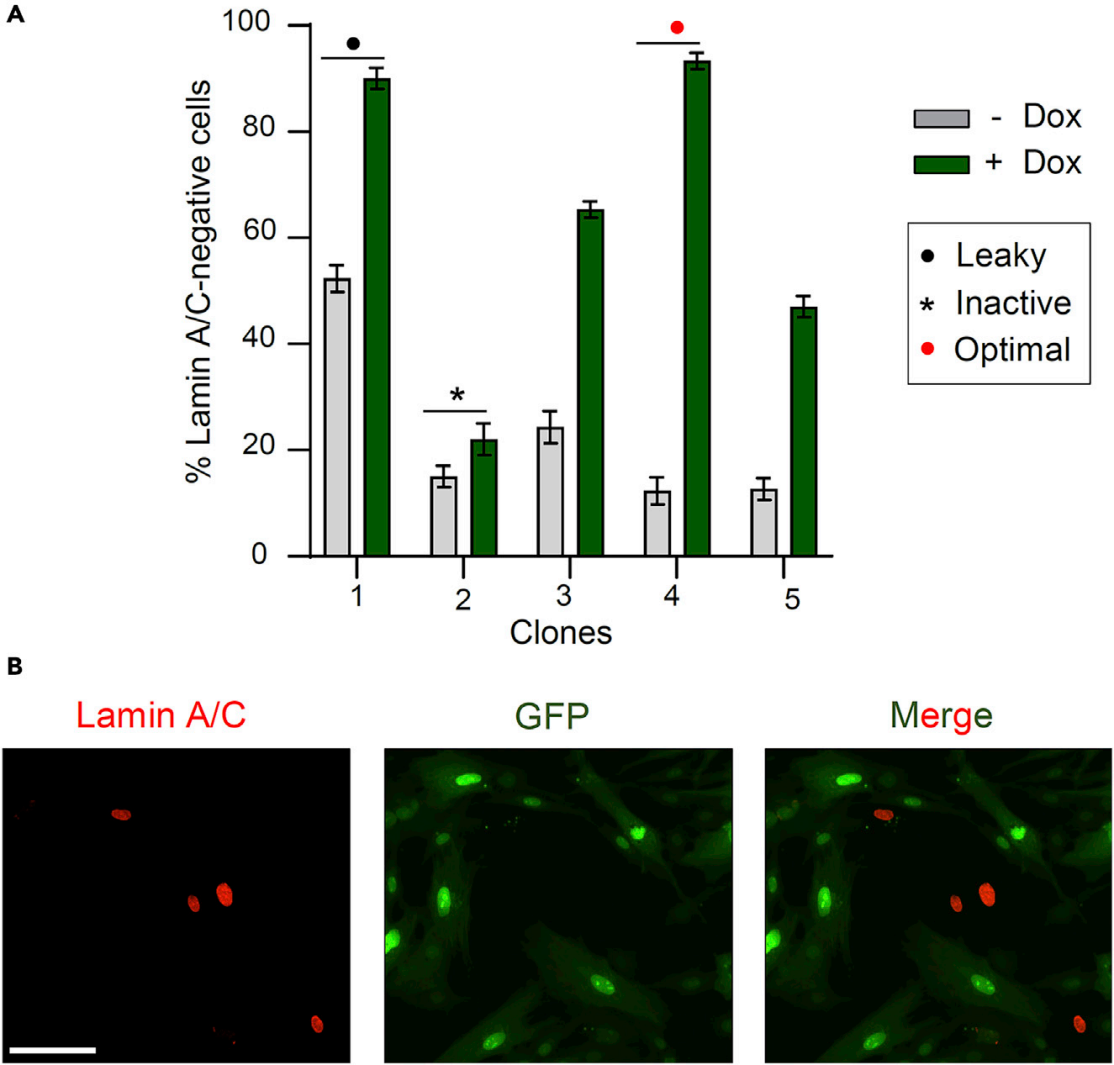
Screening of iCas9-expressing clones by editing activity (A) Quantitative immunofluorescence microscopy of cells in which KO of *LMNA* had been induced upon doxycycline treatment. Cells without doxycycline treatment are used to assess leakiness of Cas9 expression. Data are presented as mean ± SEM. (B) Immunofluorescence microscopy of doxycycline-induced clone 4 cells expressing *LMNA*-targeting sgRNA. Scale: 100 μm.

***Note:*** The percentage of Lamin A/C-negative cells is used as a proxy for KO efficiency, and is controlled for infection efficiency as only GFP + cells are analysed. Optimal clones are selected based on maximal fraction of Lamin A/C-negative cells in dox-treated cells (high activity) and minimal fraction in untreated cells (low leakiness of the Cas9 construct) (Figure 1).

7. Expand clonal cell line and freeze/store for future use.

### Generation of arrayed KO populations via lentiviral transduction of sgRNAs – Option 1

**Timing: 15 days**

The purpose of this step is to produce the arrayed library of lentiviral particles using HEK293T cells. This protocol begins from having the plasmid library in the desired plate layout as described in the “plate layout in 96-well format” section. The virus production protocol is the same as pLenti-sg*LMNA*-GFP virus production but it has been optimized for large-scale use in 96-well plates.

8. Optimized conditions for a 96-well format. Prepare lentiviral plasmid mix for transfection as below:

**Table:** 

Reagent	Amount
pLenti sgRNA (library plasmid)	67.5 ng
psPax2	50.6 ng
pMD2G	16.8 ng
pAdvantage	15 ng
OptiMEM	35.45 μL
FugeneHD	0.45 μL
  a. Leave for 15 min at 20°C–25°C.
  b. Add the transfection mix to each well of HEK293T cells as per library design.
  c. Recover the supernatant containing the viral particles, and filter through a 96-well filter plate with 0.45 μm pore size.
    ***Note:*** This step can be done 24 h post transfection and repeated at 48 h for the second batch of virus.
  d. Seal plates containing the viral particles and store them in aliquots at −80°C.
    ***Note:*** pAdvantage is a plasmid that increases translation initiation in cells transiently transfected with protein expression constructs. It is not an essential component of the system but facilitates the production of high-titer virus.
    The library of lentiviral particles will now be used to transduce Cas9-expressing MEXF 2090 cells and generate arrayed KO populations.

9. Day1: MEXF 2090 seeding.
  a. Culture MEXF 2090 cells in complete RPMI medium containing 10% FBS and 1% Pen/Strep.
  b. Take one 10-cm dish of healthy cells (confluency: 70%–80%), trypsinize and count.
  c. Seed cells in 96-well plates (5,000 cells/well) to reach ∼30%–50% confluency for the following day.
10. Day 2: Infection.
  a. Remove medium by aspirating.
  b. Dilute the generated viral supernatant (1:8) in RPMI and add 5 μg mL^−1^ polybrene and add to cells, at 100 μL per well.

***Note:*** Viral dilution needs to be established depending on the cell line used and the virus titer. Use the maximum dose of the virus that does not lead to cell death or affect cell proliferation in the negative control wells.

***Note:*** Unlike approaches employing pooled sgRNA libraries, low multiplicity of infection (MOI) and expression of single sgRNAs in individual cells is not a requirement for the success of the experiment. As long as KO is efficient and toxic MOI is avoided, the final outcome is independent of the achieved MOI.

11. Day 3–8: Selection with blasticidin.
  a. 24 h post infection, treat MEXF2090 cells with 5 μg mL^−1^ blasticidin.

***Note:*** Selection is discontinued when control uninfected wells present total death (4 days). If needed, split cells in between by maintaining the blasticidin selection. Blasticidin concentration and time under selection can vary among different cell lines, and needs to be defined before starting.

**Figure 2:**
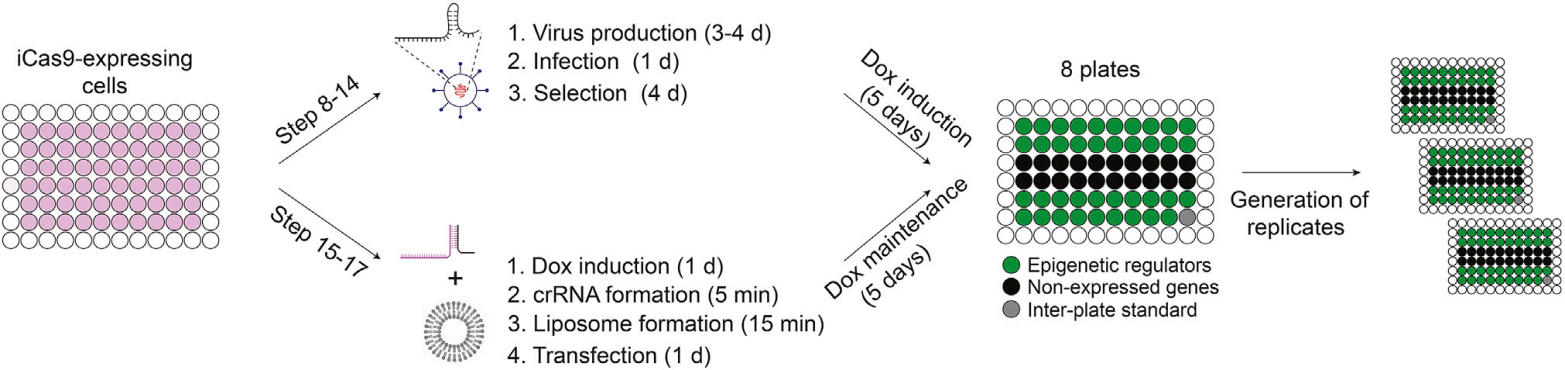
Generation of arrayed KO populations Schematic representation of the generation of arrayed KO populations via lentiviral transduction of sgRNAs or via transfection of synthetic crRNAs. Figures adapted from Loukas et al.^1^

12. Day 9: Freezing cells in 96-well plates.
  a. Discard the medium by inverting the plate.
  b. Add 30 μL of trypsin and leave the plate in the incubator for 4 min.
  c. Add 80 μL of ice-cold freezing medium (90% FBS + 10% DMSO).
    ***Note:*** Freezing medium can vary among cell lines.
  d. Seal the plate and store it at −80°C.
  e. To thaw cells, place plates in a water bath at 37°C for a few seconds, add 50 μL of medium to each well and spin down at 300 g for 5 min at 4°C. Remove 120 μL of liquid and add 100 μL of fresh medium to each well.
    **Pause point:** An aliquot of selected MEXF 2090 cells can be frozen at this point and stored at −80°C for future usage.

13. Day 10: Generation of KO cell populations – Cas9 induction via doxycycline treatment.
  a. Replace the cell with medium containing 1 μg mL^−1^ doxycycline. Incubate for 5 days.
14. Day 15: The arrayed KO populations are ready to be used for the large-scale fitness assay (move to step 18) (Figure 2).

### Generation of arrayed KO populations via transfection of synthetic crRNAs – Option 2

**Timing: 7 days**

The arrayed collections of predesigned synthetic crRNAs will now be used to transfect Cas9-expressing MEXF 2090 cells.

15. Day 1: Cas9 induction.
  a. Culture MEXF 2090 cells in complete RPMI medium containing 10% FBS and 1% Pen/Strep. Take two 10-cm dish of healthy cells (70%–80% confluency) and add 1 μg mL^−1^ doxycycline to induce Cas9 expression.

16. Day 2: Transfection.
  a. Resuspend the 0.1 nmol crRNA library in 5 μL/well of 1× siRNA buffer (which is diluted from 5× siRNA Buffer (Dharmarcon) with DNAse/RNAse free water) for a 20 μM stock.
  b. Resuspend 20 nmol trRNA in 1 mL for a 20 μM stock.
  c. Add to each well of the 96-well plate library 5 μL of trRNA and incubate for 5 min at 20°C–25°C to allow RNA annealing.
  d. Dilute crRNA/trRNA into 500 μL using OptiMEM to make 200 nM. Aliquot library onto PCR plates (100 μL each) and store at – 20°C for future usage.
    **CRITICAL:** 20 μM stocks must be used to form the crRNA/trRNA complex. Diluted molecules (for instance addition of 500 μL of 200 nM trRNA to individual 20 μM crRNAs) will result in inefficient complex formation and gene KO.
  e. Prepare diluted transfection reagent for all needed conditions, with a little excess (0.3 μL DharmaFECT Duo per 10 μL OptiMEM /96-well for MEXF 2090 cells).
  f. Prepare new 96-well plates according to how many transfections you need to perform.
    ***Note:*** Fitness assays should be done at least in triplicate for each condition (untreated vs treated).
  g. For each condition move 10 μL of the diluted crRNA library onto a new 96-well plate and add 10 μL diluted transfection reagent to each well, mix and incubate for 15–20 min.
  h. Retrieve Cas9-expressing cells from the incubator. Trypsinize and count cells.
  i. Calculate the necessary volume of cell suspension for 4,000 cells per well in 80 μL complete medium with doxycycline.
  j. Add 80 μL of cell suspension per well in the 96-well plate. Final concentration of cRNA/trRNA mix in the well will be 20 nM.
  k. Culture cells for 5 days and expand as necessary, adding doxycycline for at least 5 days, at which point editing should be complete.

17. Day 7: The arrayed KO populations are ready to be used for the large-scale fitness assay (Figure 2).

**CRITICAL:** The transfection reagent should be optimized for the cell line used. Difference in transfection efficiency could lead to major changes in the percentage of KO cells within the population. Potential transfection reagents to test using *LMNA*-targeting crRNAs and the immunofluorescence assay described in the section above are: Lullaby, DharmaFECT Set of 4 Transfection Reagents, DharmaFECT Duo and Lipofectamine RNAiMAX.

### Large-scale fitness assays

**Timing: 2 weeks**

In the large-scale experiment, sufficient identical replicates for the different treatments, are generated from each library plate. From a confluent arrayed KO plate (step 16 – generation of arrayed KO populations via lentiviral transduction of sgRNAs – option 1; or step 3 – generation of arrayed KO populations via transfection of synthetic crRNAs – option 2), cells are split at an empirically-defined ratio to achieve approximately 2000 cells – in our case 1:30. Then, cells are either grown under stressful conditions or maintained in unperturbed conditions. Over the course of the experiment one representative plate is monitored to confirm that the growth kinetics is similar to the one expected based on the stress titration pilot experiments, and also dictates the respective endpoint for each stress where the cells will be quantified by nuclei measurement.

***Note:*** The endpoint depends on how severely cells are affected by each stress, with glutamine deprivation being the most deleterious stress; it is determined by when the fittest population approaches confluency, varying from 3 d (acidity and Buparlisib) to 7 d (glutamine deprivation). Unperturbed cells are fixed 2 d after seeding, and allow normalization across wells, accounting for small differences in numbers of seeded cells, as well identification of stress-specific effects. At the indicated endpoints for each condition, the population fitness is assessed by quantification of cell count.

***Note:*** Before proceeding to the remaining part of the protocol, it is important to check KO efficiency in one of the replicate plates. Select 2–3 genes, whose antibodies are available and of good quality, and perform standard immunofluorescence. The sgRNA library used in this protocol has shown overall high activity.^8^

18. DAY 0: Seeding cells.
  a. For each plate, discard medium by aspirating and gently wash with PBS.
  b. Add 30 μL of Trypsin and incubate for 5 min.
  c. Add 170 μL of complete RPMI and pipette to resuspend.
  d. Transfer the whole suspension of cells to a 2 mL deep well block and mix with fresh RPMI medium to achieve the desired dilution.
  e. Transfer 50 μL of cells into new plates containing 50 μL of fresh media. Gently shake the plate to ensure homogeneous plating.

***Note:*** The above dilution ratios and the volumes of seeded cells and media within the recipient wells are indicative and can be tuned to achieve different scales of seeding (e.g., plating 10 replicate plates vs 30 replicate plates).

19. DAY 1: the median cell counts per well across the 96-well plates should be around 4,000. At this step, the plates are grown in the following conditions: a) unperturbed b) glutamine deprivation (0% L-Glutamine) c) acidic environment (pH 6.5) and d) PI3Ki (1nM Buparlisib).
  a. Discard medium by aspirating and gently wash with PBS.
  b. Add 100 μL per well of RPMI complete or RPMI with the respective stressor.
20. DAY 2–7: Buparlisib treatment and media acidification is sustained for 3 days, while glutamine deprivation is sustained for 7 days.

***Note:*** Cell count for populations grown in unperturbed conditions is quantified at day 2, which at that day results in a 40%–60% reduction in cell counts compared to untreated cells in negative control KO populations.

**CRITICAL:** It is essential to keep the outer wells filled with PBS for optimal humidity levels of the plates. If that it is not done, cells close to the edge suffer and die, especially when experiencing harsh conditions.

21. At the indicated endpoints for each condition, fix plates with 4% PFA followed by nuclei staining with SYTOX Green or Red.

### Imaging and data analysis

**Timing: 1–2 weeks**

**Figure 3:**
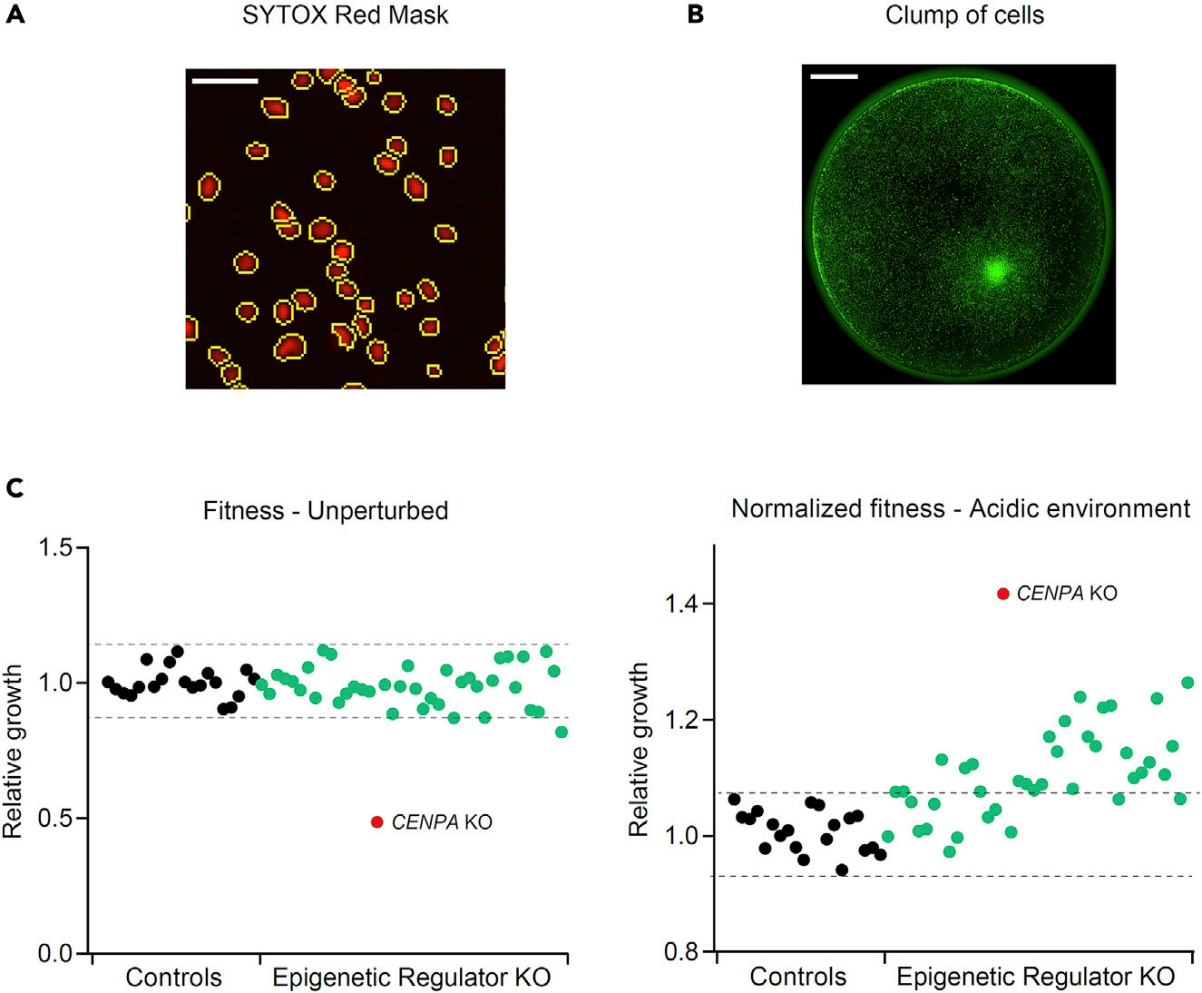
Assessment of possible technical artefacts (A and B) Representative pictures of SYTOX Red-stained nuclei (red) surrounded by the Incucyte mask (yellow) (A) and of a cell clump in one well (B). Scale bars: 100 μm and 1 mm, respectively. (C) Example of inflated normalized fitness due to detrimental effects of gene KO in unperturbed conditions, rather than a true stress-specific effect (*CENPA*-KO). Values represent the growth of individual epigenetic regulators KO populations relative to the negative controls from one of eight plates, grown in the indicated conditions. Dashes indicate growth range of control populations.

***Note:*** Default settings are typically suitable to create an accurate mask and identify nuclei. However, the mask can be optimized by changing parameters such as cell size to exclude background speckles.

22. Imaging and quantification are performed in Incucyte® S3 Live-Cell Analysis System.
  a. Scan type: whole well (96-well plate format).
  b. Scan settings: Acquisition time: 300 ms (SYTOX Green), 400 ms (SYTOX Red).
23. Once the plates are scanned, use default settings to identify nuclei and quantify cell counts (Figure 3A).

24. Quality control via manual inspection of images is essential. Various parameters must be assessed to ensure that the data is of sufficient quality before quantification of fitness:
  a. Wells containing cell clumps must be removed; it would strongly affect the measurement (Figure 3B).
  b. Outliers among replicates may be removed (typically differing from the two consistent replicates more than 3 times their difference).
  c. KO populations with severely compromised fitness in unperturbed conditions must be removed. In such cases, the linearity of comparison is lost and the normalization (growth under stress/growth under normal conditions) artificially confers an apparent fitness advantage to populations with decreased growth kinetics (20% reduction compared to the plate median – Figures 3C and 3D).

***Note:*** As an example, in Loukas et al.,^1^ 68 genes were excluded from the analysis because they conferred a severely compromised fitness under unperturbed conditions; leaving 250 cell populations. Moreover, 61 out of 7200 total imaged wells (0.85%) were excluded from the analysis because of poor-quality data: 53 wells (87% of excluded wells) because of visible clumps of cells and the remaining 8 wells because they were highly inconsistent with the other two replicates (typically differing from the two consistent replicates more than 3 times their difference).

**Figure 4:**
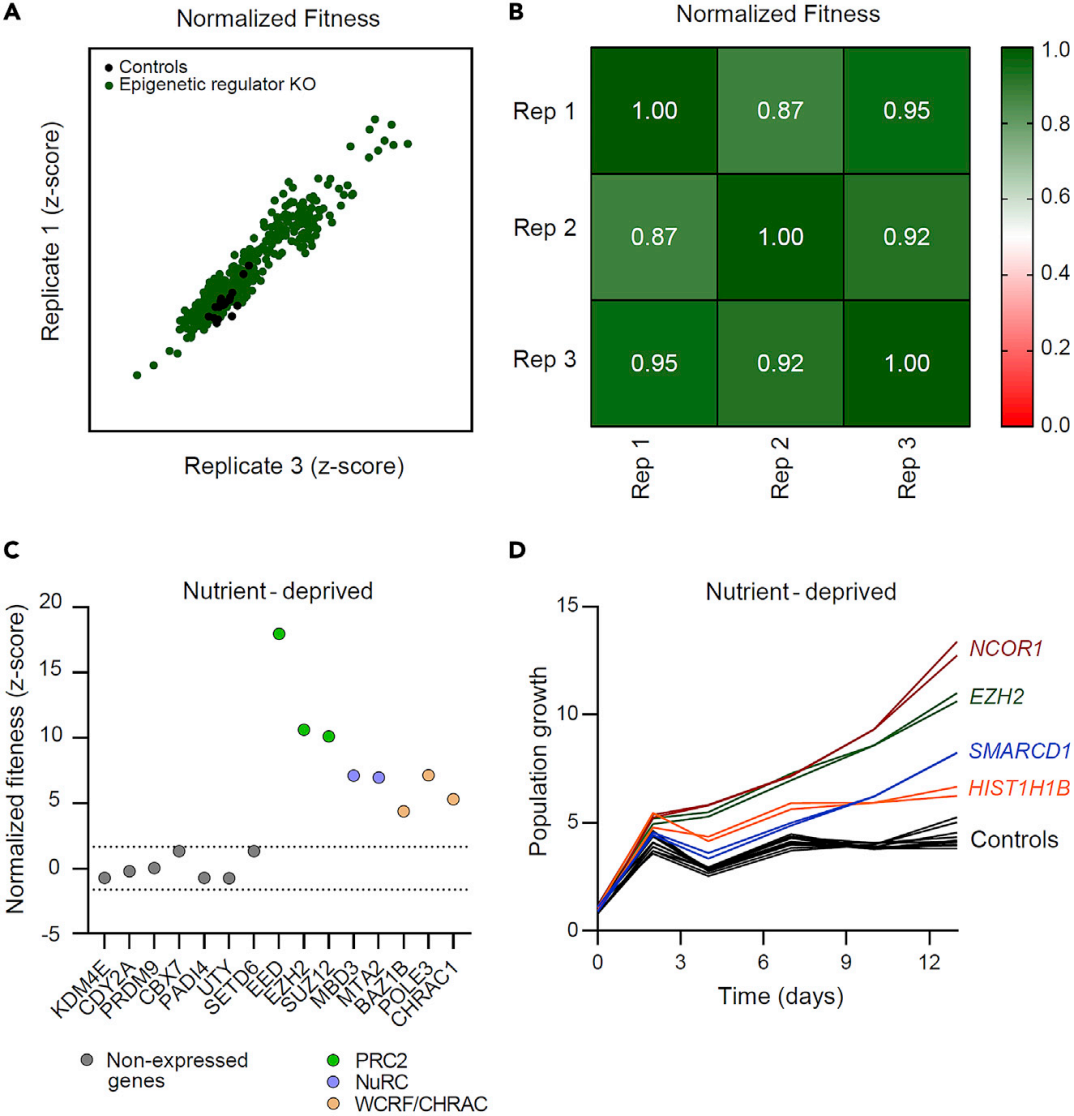
Reproducibility of the data generated by the large-scale CRISPR screen (A) Correlation between two biological replicates of MEXF 2090 cells grown under glutamine deprivation. (B) Table of the r squared values of the indicated biological replicates. (C) Quantification of normalized fitness for the indicated KO populations of MEXF 2090 cells. Members of the same protein complex are color-coded. The dashed lines indicate the z-score threshold values of neutral phenotypes defined by negative controls. (D) Growth kinetics of 4 KO and 5 control populations of MEXF 2090 cells selected for validation of the results from the large-scale assay. Two independent biological replicates are shown. Figures adapted from Loukas et al.^1^

25. Results quantification.
  a. Use the stress/unperturbed ratio in cell count at endpoint to calculate the normalized fitness for each KO or control population.
  b. Define KO populations with enhanced or reduced fitness based on the formula: Z=(χ-μ)/σ, where χ is the fitness of individual KO populations, μ is the mean fitness of negative controls, σ is the standard deviation of the fitness of negative controls.
  c. Define populations exhibiting enhanced or reduced fitness as those with a z-score > 1.645 or < -1.645 (90% confidence interval), respectively. Since a high ratio of sample vs negative controls (2:1) is used and the z-score includes information on confidence level, correction for multiple comparisons is not required.
  d. Perform validation experiments with selected genes exhibiting varying degree of stress-resistance, including mild phenotypes. An example is shown in Figure 4.

## Expected outcomes

Following this protocol, a sgRNA or a crRNA library targeting a selected gene network is sourced and arranged in a 96-well plate format to induce its systematic disruption. The effect of this network-level perturbation on cell fitness under various stressful conditions is then assessed. When following the protocol as described, expected outcomes include high reproducibility (Figure 4A), with high correlation between biological replicates in different plates (above 0.8, Figure 4B), and similar phenotypes upon KO of members of the same protein complexes (Figure 4C). This highlights the success and the robustness of the large-scale arrayed assay. Moreover, lowly- or non-expressed genes do not exhibit any deviating phenotype from the norm in any condition, ruling out off-target effects. Based on this initial interrogation of the data, validation experiments were performed and demonstrated high reproducibility rate.^1^ The arrayed format of the assay along with the high reproducibility among replicates allows detection of significant but subtle phenotypes that could have been missed via other means of experimental interrogation (e.g., selection-based pooled CRISPR screens, Figure 4D). The detection of similar phenotypes within members of the same complex suggests that the generated dataset can be used as a resource to detect a) putative interactors b) *de novo* functional links between proteins of seemingly unrelated functions. On top of that, this approach allows exploration of fitness relationships and patterns at multiple scales depending on the biological question under examination (i.e., impact of individual genes in multiple stress conditions, and interrogation of entire gene classes of related function).

## Limitations

Despite the selection of transduced cells by antibiotic treatment or the high transfection efficiency of crRNAs, gene KO may not occur in all cells within the population due to in-frame in/dels. Thus, estimation of the KO efficiency during the generation of the libraries is needed to interpret the results and the phenotypic differences. In fact, the presence of a significant percentage of wild-type cells within the edited population leads to an underestimation of the actual differences between KO and control populations. Moreover, depending on the genetic background of the chosen line, some detected phenotypes may be synthetic interactions specific to the line. Performing the described functional assays in multiple models is needed to draw general conclusions.

The success of the approach relies on reliable measurements of cellular features by microscopy. Here, we have stained nuclei to count cell numbers and infer cell fitness, but this protocol can be applied to any microscopy-based readout, provided that the dynamic range of the assay allows detection of mild phenotypes and that reagents used (e.g., antibodies) are of good quality. Cell number can also be estimated by colorimetric assays as performed in Moreno et al,^9^ although the sensitivity of these approaches is lower.

This protocol can be adapted for use in various cell lines, employing custom guide RNA libraries and testing different external or internal stressors.

## Troubleshooting

### Problem 1

Decrease in the KO efficiency of the generated Cas9 clone over time (step-by-step method details, step 7).

### Potential solution

Cell fitness can be mildly affected by Cas9 expression, even at basal levels in uninduced cells; thus epigenetic silencing of the transgene may be favorable and selected over time, leading to subsequent reduction of the editing efficiency. Avoid prolonged culture and generate a large number of identical frozen stocks of the successful clone. Moreover, periodical re-selection with hygromycin may prevent or solve such complications.

### Problem 2

Low efficiency during HEK293T cells transfection (step-by-step method details, step 14).

### Potential solution

Always use a GFP-expressing lentiviral construct along the generation of the arrayed KO lentiviral library (e.g., pLenti-sg*LMNA*-GFP). The visual inspection of GFP positive cells will aid the step-by-step troubleshooting, revealing the exact procedure that was not optimal (transfection, infection, etc).

Possible causes:

• overconfluent or sparsely seeded HEK293T cells; optimize number of seeded cells.
• suboptimal transfection conditions; alternative transfection reagents to be considered.

### Problem 3

Low infection efficiency during the generation of the KO library (step-by-step method details, step 14).

### Potential solution

• If toxicity is observed, refresh media containing the viral particles 5–6 h post infection and/or decrease titer.
• if toxicity is not observed increase to maximal MOI that does not lead to cell death and/or perform another round of infection to the cells (in consecutive days).
• If frozen viral aliquots are used, avoid repeated freeze/thaw cycles.

### Problem 4

Dependency of stress conditions on initial number of cells leading to substantial technical noise and confounding variability between KO populations (step-by-step method details, step 24).

### Potential solution

Some stressors are strongly dependent on the cell density. This translates to cases where the same population, that theoretically should show a similar response, exhibiting different fitness depending on the initial density. This dependency becomes increasingly problematic in large scale assays, which are inherently characterized by such variability in this first step (number of seeded cells based on uniform splitting ratio across different KO populations). Glutamine starvation is not strongly dependent on cell number, but others are, such as oxidative stress by treatment with hydrogen peroxide. Therefore, it is crucial to assess whether the initial response to the applied stress is dependent on the starting cell number. To do so, plate varying densities of control populations and explore the linearity of their response within a range that mimics the extent of variability expected in large scale CRISPR assay (∼ 20%–30% deviation from the intended cell count).

If the stress is strongly dependent on cell count two possible modifications of the current protocol exist, where the choice mainly depends on the scale of the experiment:

• Large-scale: Use 96-well plates that contain by design varying initial densities (400–500 cell increments) of the control population. Pre-treatment confluence imaging of both the KO library and the control plate. At the endpoint compare the phenotypes in each KO and normalize against a bin of control cells that had similar starting density.
• Low scale: normalize cell count before application of stress. To do so, 1–2 days before the experiment create a replicate plate (1:2 splitting). Before the application of stress quantify cells on the replicate plate by IncuCyte imaging and based on that alter the seeding volumes to normalize the starting density across populations.

### Problem 5

Presence of cell clumps (step-by-step method details, step 24).

### Potential solution

Always use fresh trypsin and avoid dispensing it directly onto the cells, as this can create big clumps that are difficult to dissociate even after vigorous pipetting.

## Resource availability

### Lead contact

Further information and requests for resources and reagents should be directed to and will be fulfilled by the lead contact, Paola Scaffidi (paola.scaffidi@crick.ac.uk).

### Materials availability

The epigenetic sgRNA library and lentiviral constructs to transduce Cas9 and *LMNA*-targeting sgRNAs described in this study are available upon request. MEFX 2090 cells are covered by an MTA and cannot be transferred.

## Data Availability

Z-score tables from the large-scale fitness assays described in Loukas et al.^1^ are available as supplemental data of the cited article (Table S3 of Loukas et al.).
